# Association between functional movement quality, core stability, and athletic performance in male university basketball players

**DOI:** 10.3389/fspor.2026.1813066

**Published:** 2026-08-27

**Authors:** Yu Xuedou, Ma Qiyuan, Kang Dongwei, Geng Jin

**Affiliations:** 1 Hebei University of Engineering, School of Sports and Health Engineering, Handan, China; 2 Hebei University of Engineering, School of Management Engineering and Business, Handan, China

**Keywords:** athletic performance, core stability, correlation, functional movement, university basketball players

## Abstract

To provide empirical evidence for the specialized training and injury prevention of male university basketball players, this study aims to investigate the correlations among core stability, functional movement quality, and basketball-specific performance, elucidating the independent contributions of functional movement patterns and core endurance to athletic performance. Thirty-three healthy male university basketball players were recruited. Core stability was assessed using McGill's trunk core endurance tests (i.e., trunk flexor, trunk extensor, left-side bridge, right-side bridge), functional movement quality via the Functional Movement Screen (FMS; including deep squat, hurdle step, in-line lunge, shoulder mobility, active straight leg raise, trunk stability push-up, and rotary stability), and basketball performance through specific tests: vertical jump, shooting, and change-of-direction dribbling. Pearson correlation analysis was employed to examine relationships between variables. The study found that functional movement levels had a strong correlation with vertical jump performance, but their associations with shooting and change-of-direction dribbling were weak or non-significant. Core muscle endurance, particularly of the back extensors, was strongly correlated with high-intensity dynamic change-of-direction performance, and performance was influenced by left-right asymmetry in lateral core endurance. Core stability and functional movement patterns were not significantly correlated in this study, suggesting they may be independent in structure and function. Based on these correlation results, subsequent training may consider optimizing functional movement patterns and strengthening core stability separately, to explore their potential effects on performance enhancement and injury prevention.

## 1 Introduction

Basketball is a team sport characterized by high-intensity intermittent sprints, complex technical skills, and rapid decision-making. Sport-specific actions such as changing pace and direction, jumping for shots, and finishing through contact are often executed under dynamic imbalance. The quality of these actions is highly dependent on core stability (1) and the synergy of functional movement (2).

From a theoretical mechanism perspective, McGill emphasizes the foundational role of core stability, positing that the fatigue resistance of trunk musculature forms the physiological basis for dynamic stability. The core muscles act as a pivotal hub in the kinetic chain, regulating intra-abdominal pressure and thoracolumbar fascia tension to establish an energy-efficient pathway for force transmission from the limbs (3), directly influencing performance efficacy and injury risk (4, 5). Conversely, Cook's functional pyramid model advocates that optimizing movement patterns enhances performance, designating the Functional Movement Screen (FMS) as the gold standard for assessing movement quality, suggesting that imbalances in flexibility, stability, and symmetry can limit performance and increase injury risk (2). Existing research often explores the relationship between either functional movement or core stability and athletic performance, overlooking their combined influence on basketball performance. Considering the specific characteristics of basketball, recent studies have further revealed the critical role of balance, stability, and neuromuscular control. Basketball involves frequent sagittal plane jumping and frontal plane directional changes, requiring core stability to both provide rigid support for transmitting lower limb force to the upper body and to maintain movement quality under dynamic perturbation (6–9). The FMS assesses comprehensive performance in flexibility, stability, symmetry, and coordination through seven fundamental movement patterns and four clearing tests, thereby predicting and evaluating athletic performance and injury risk (10–14).

Current research predominantly investigates the relationship between either functional movement or core stability and performance in isolation, lacking focus on their synergistic effect. Notably, Okada et al. (15) found only weak correlations between core stability, FMS, and performance, with no significant link to FMS, though their testing format did not adequately reflect the specific technical characteristics of basketball, leading to limitations in their conclusions. There remains a lack of empirical research specifically targeting male university basketball players to clarify the independent contributions of core stability and functional movement quality to different aspects of athletic performance, which constitutes the core research gap addressed by this study.

Based on this, This study proposes that core stability and functional movement quality are two independent evaluation dimensions, with differential associations with basketball performance. Core endurance may correlate strongly with change-of-direction and jumping ability, while functional movement patterns may correlate with jumping and technical execution quality.

This study aims to clarify the correlations among core stability, functional movement quality, and basketball-specific performance through quantitative analysis, elucidating their independent contributions to athletic performance, thereby providing empirical evidence for coaches to develop scientific training programs, enhance performance, and prevent sports injuries.

## 2 Methods

### 2.1 Study design

This study employed a cross-sectional observational design, utilizing standardized testing and correlation analysis to investigate the associations and independent contributions of core stability, functional movement quality, and basketball-specific performance in male university basketball players. All tests were completed at the Sports Training Laboratory of Hebei University of Engineering, with testing procedures strictly adhering to ethical norms for human experimentation and sport science testing standards.

### 2.2 Sample size and selection

This study strictly followed the inclusion and exclusion criteria (see Table 1). A total of 33 healthy male university basketball players were recruited, all of whom were students from the Department of Sports Training, School of Sports and Health Engineering, Hebei University of Engineering, and volunteered to participate in the study. The study was approved by the Biomedical Ethics Committee of School of Medicine, Hebei University of Engineering (Ethics Approval Number: YXY-2025069). The study involved human subjects, and all testing procedures strictly followed ethical guidelines. All 33 participants signed written informed consent forms, which clearly explained the testing procedures, risks, and research purpose.

**Table 1:** Inclusion and exclusion criteria for study participants.

Category	Specific criteria
Inclusion Criteria	Sex: Male Age: 18–25 years Status: Current university basketball player Training Experience: At least 3 years of systematic basketball training Skill Level: National Level 2 athlete or higher Physical Condition: No acute sports injuries, able to complete all test movements Ethical Requirement: Signed written informed consent
Exclusion Criteria	History of musculoskeletal injury, surgery, or chronic pain within the past 6 months affecting testing Presence of cardiovascular, neuromuscular, or other systemic diseases preventing tolerance of high-intensity testing Poor physical condition on the test day (e.g., fatigue, cold) assessed as unsuitable for participation

The average training duration of the participants was 5.18 ± 0.84 years, and the average basketball experience was 6.70 ± 0.91 years (see Table 2). To assess the representativeness of the sample, these indicators (training duration and basketball experience) were compared with publicly available data from the Chinese national collegiate men's basketball player population. An independent-samples t-test revealed a statistically significant difference (t = 1.72, *P* = 0.003; see Table 3), indicating that the training duration of the sample in this study falls within the typical range of the national collegiate men's basketball player population.

**Table 2:** Basic information of participants.

Number	Name	Training Duration (Year)	Competitive Level	Years of Basketball Experience (Year)
1	Zhang**	5	National Level 2	6
2	Ji**	6	National Level 2	8
3	Bo**	4	National Level 2	6
4	Gao**	5	National Level 2	6
5	Zhao**	4	National Level 2	5
6	Zhou**	3	National Level 2	5
7	Ma*	4	National Level 2	6
8	Huang**	6	National Level 1	8
9	Geng**	6	National Level 1	8
10	Liu**	5	National Level 2	7
11	Wang**	6	National Level 2	6
12	Li**	6	National Level 2	6
13	Song**	5	National Level 2	7
14	Xue**	6	National Level 2	7
15	Dai**	5	National Level 2	6
16	Sun**	6	National Level 2	6
17	Li*	4	National Level 2	5
18	Zhao**	6	National Level 2	6
19	Jing*	5	National Level 2	7
20	Wan**	4	National Level 2	7
21	Su**	5	National Level 2	6
22	Wang**	5	National Level 2	6
23	Wang**	4	National Level 2	5
24	Tong**	6	National Level 2	6
25	Ma**	5	National Level 2	7
26	Sun**	4	National Level 2	5
27	Chen**	6	National Level 2	6
28	Li**	5	National Level 2	7
29	Wang**	5	National Level 2	6
30	Zhou**	6	National Level 2	7
31	Wang**	5	National Level 2	6
32	Fu*	4	National Level 2	7
33	Zhang*	5	National Level 2	7

**Table 3:** Independent-samples T-test.

Independent-Samples t-Test		Levene's Test for Equality of Variances		t-test for Equality of Means						
		F	Sig.	T	df	Sig. (2-tailed)	Mean Difference	Sth. Error Difference	95% Confidence Interval of the Difference	
									Lower	Upper
Training duration (years)	Equal variances assumed	2.596	0.117	1.72	31	0.002	1.032	0.6	−0.191	2.256
	Equal variances not assumed			6.875	30	0	1.032	0.15	0.726	1.339

### 2.3 Testing procedures

This study followed a testing sequence starting with the Functional Movement Screen, followed by core stability tests, and concluding with basketball-specific performance tests. This order follows the scientific principle of progressing from basic functional assessment to sport-specific evaluation, and from low-intensity to high-intensity tasks.

The FMS consists of seven low-load, bodyweight-based pattern movements, each typically repeated three times. The entire FMS procedure is short (approximately 10–15 min) and imposes minimal physiological demand, resulting in negligible metabolic or neuromuscular fatigue that could affect subsequent high-intensity tests. Therefore, conducting the FMS first does not interfere with later tests due to fatigue or carryover effects, but rather ensures that authentic baseline functional data are obtained. Placing the FMS after high-intensity tests would allow fatigue to compromise its screening effectiveness.

The recommended rest intervals between tests are as follows: 5–10 min of rest after the FMS; 3–5 min of rest after the standardized warm-up (to take advantage of the activated state while avoiding fatigue); and adequate rest between the core stability tests and the sport-specific performance tests. This sequence and rest schedule ensure that all tests are performed when participants are in their respective optimal states, thereby enhancing data reliability and validity.

The final testing sequence consisted of: (1) FMS screening, (2) 5–10 min rest, (3) standardized warm-up, (4) 3–5 min rest, (5) core stability tests, (6) adequate rest, and (7) sport-specific performance tests.

Before the formal test, professional testers provided unified instructions and demonstrations, and a pre-test practice session was conducted. After the formal test began, participants first completed the Functional Movement Screen. Subsequently, they underwent a 15-minute standardized dynamic warm-up and specific activation, followed by the remaining two tests, with a 10-minute active rest period between them. All tests were completed in the indoor basketball gymnasium and Sports Function Laboratory of Hebei University of Engineering, with the ambient temperature controlled at 22–25 °C. Testing times were uniformly scheduled between 14:00 and 17:00. The testing procedures were strictly standardized to ensure data reliability and reproducibility.

### 2.4 Instruments and measurements

#### 2.4.1 Core stability test

Core stability was assessed using the McGill-designed trunk core stability tests, comprising tests for trunk flexor endurance, trunk extensor endurance, left trunk endurance and right trunk endurance.

##### 2.4.1.1 Trunk extensor endurance test

This test evaluates the muscular endurance of the trunk extensors (i.e., erector spinae, longissimus, iliocostalis, and multifidus).

Method: The participant assumed a prone position with the iliac crests aligned with the edge of a bench. The upper body was supported off the bench, with arms on the floor or a platform. While the subject supports his/her upper body weight, nylon straps are used to secure the subject's lower legs to the bench. If straps are not available, the tester stabilizes the subject's legs using his/her own body weight. The objective of the test is to maintain a horizontal prone position for as long as possible. The test is terminated as soon as the subject falls below the horizontal position. After the subject is prepared, he/she lifts/extends the trunk until it is parallel to the ground, with arms crossed over the chest. Immediately upon achieving this position, the stopwatch is started. When the subject can no longer maintain the position, the test is terminated, and the duration for which the subject held the position is recorded on the test form.

##### 2.4.1.2 Trunk flexor endurance test

This test assesses the muscular endurance of deep core muscles (i.e., transversus abdominis, quadratus lumborum, and erector spinae).

Method: During the test, the subject begins in a seated position with hips and knees bent at 90 degrees, ensuring that the hips, knees, and second toes are aligned. The subject is instructed to cross the arms over the chest, with each hand touching the opposite shoulder, while leaning against an inclined training board set at a 60-degree angle. The head should remain in a neutral position. It is essential that the subject presses the shoulders firmly against the board and maintains this “open” posture after the board is removed throughout the entire test. The subject is instructed to tighten the abdominal muscles to keep the spine in a flat neutral position. The back must not arch during the test. The tester may secure the subject's toes under a training strap or, if necessary, stabilize the feet with hands. The objective of the test is to maintain this 60-degree position without back support for as long as possible. The stopwatch is started when the tester removes the board by approximately 4 inches (10 cm), while the subject maintains the 60-degree unsupported posture. The test is terminated when a clear change in trunk posture occurs, and the duration for which the subject held the position is recorded on the test form.

##### 2.4.1.3 trunk lateral endurance test

The Trunk Lateral Endurance Test, commonly referred to as the Side Bridge Test, assesses the muscular endurance of the lateral core muscles (i.e., the transversus abdominis, obliques, quadratus lumborum, and erector spinae).

Method: During the test, the starting position requires the subject to lie on their side with legs extended, stacking one foot on top of the other or positioning the feet in a heel-to-toe placement. The subject places the lower arm beneath the body and the upper arm along the side of the body. Once prepared, the subject is instructed to assume a full side bridge position, keeping the legs straight and the lateral sides of the feet in contact with the ground. The elbow of the lower arm should be positioned directly under the shoulder, with the forearm pointing outward (the palm can face down for balance and support). The upper arm should be placed along the side of the body or across the chest to rest on the opposite shoulder. The hips should be lifted off the mat, and the body should form a straight line (i.e., head, neck, torso, hips, and legs). The torso should be supported only by the subject's foot/feet and the elbow/forearm of the lower arm. The objective of the test is to maintain this position for as long as possible. The tester starts the stopwatch when the subject is in the side bridge position. The test is terminated as soon as the subject breaks the position. The duration for which the position was held is recorded on the test sheet. The test is repeated on the opposite side, and the value is recorded accordingly.

#### 2.4.2 Functional movement test

Functional movement was assessed using the Functional Movement Screen (FMS), which consists of seven test movements and four exclusion tests. The final score was determined based on the results of the seven primary movement tests and the four exclusion tests. The movement patterns used in the seven tests are all basic, measurable movements that reflect the participants' flexibility, stability, symmetry, and overall movement quality. See Table 4 for details.

**Table 4:** Functional movement screen (FMS) scoring criteria.

Test	3 Points	2 Points	1 Point	0 Points
Deep Squat	The torso is parallel or nearly vertical to the tibia The femur is below the horizontal plane The knees are positioned directly over the feet The long pole remains horizontal and aligned over the feet	The upper torso is parallel or nearly vertical to the tibia The femur is below the horizontal plane The knees are positioned directly over the feet The long pole remains horizontal and aligned over the feet	The tibia and torso are not parallel The femur is not below the horizontal plane The knees cannot be maintained directly over the feet The long pole is not aligned over the feet	Pain reported during the test
Hurdle Step	The hip, knee, and ankle maintain alignment in the sagittal plane There is minimal movement of the lumbar spine The long pole and hurdle remain parallel	The hip, knee, and ankle fail to maintain alignment in the sagittal plane There is movement of the lumbar spine The long pole and hurdle are not parallel	The hurdle rope is not cleared The body loses balance	Pain reported during the test
Inline Lunge	The long pole maintains continuous contact with the body The long pole remains vertical There is no movement of the torso The long pole and both feet stay within the same sagittal plane The rear knee touches the center of the test board The front foot remains in the starting position	The long pole fails to maintain continuous contact with the body. The long pole does not remain vertical There is movement of the torso The long pole and both feet are not in the same sagittal plane The rear knee does not touch the center of the test board The front foot is not flat in the initial position	Unable to assume the starting posture Loss of balance, with the foot lifting off the test board Unable to complete the movement pattern	Pain reported during the test
Shoulder Mobility	The distance between the fists does not exceed one hand length	The distance between the fists does not exceed one and a half hand lengths	The distance between the fists exceeds one and a half hand lengths	Pain reported during the test
Active Straight Leg Raise	The vertical line from the lateral malleolus falls between the mid-thigh and the anterior superior iliac spine (ASIS) The non-moving lower limb maintains a neutral position	The vertical line from the lateral malleolus falls between the mid-thigh and the knee joint line The non-moving lower limb maintains a neutral position	The vertical line from the lateral malleolus falls below the knee joint line The non-moving lower limb maintains a neutral position	Pain reported during the test
Trunk Stability Push-up	Male subjects complete one repetition with the thumbs aligned with the top of the forehead Female subjects complete one repetition with the thumbs aligned with the chin The subject lifts the body as a single unit without bending the spine	Male subjects complete one repetition with the thumbs aligned with the chin Female subjects complete one repetition with the thumbs aligned with the clavicle The subject lifts the body as a single unit without bending the spine	Male subjects are unable to complete one repetition with the thumbs aligned with the chin Female subjects are unable to complete one repetition with the thumbs aligned with the clavicle	Pain reported during the test
Rotary Stability	The hands and knees lift off the ground simultaneously The subject completes the movement pattern while maintaining the arm and leg in a straight line parallel to the board The fingers touch the lateral malleolus The knee and elbow joints achieve full extension	The hands and knees do not lift off the ground simultaneously The subject fails to maintain the arm and leg in a straight line parallel to the board The fingers touch the lateral malleolus The knee and elbow joints achieve full extension	The subject loses balance The hand fails to touch the lateral malleolus The knee and elbow joints do not achieve full extension The subject cannot return to the starting position	Pain reported during the test

#### 2.4.3 Athletic performance test

To accurately quantify the core elements of basketball-specific performance in male university basketball players and to ensure that the testing system aligns with domestic basketball talent selection standards, the three performance test indicators selected for this study strictly adhere to the “Sports Specific Test Methods and Scoring Standards for Winter Events of Sports Training Major in Higher Education Institutions (2025 Edition).” The selection of these indicators is justified by their policy compliance, sport-specific representativeness, and comprehensive validity.

##### 2.4.3.1 Basis for selecting basketball-specific performance tests

The vertical jump test measures lower-body explosive power, reflecting core competencies such as rebounding and shot-blocking under the basket. The one-minute restricted arc shooting test assesses dynamic, accurate, and stable scoring output, mirroring spatial scoring efficiency in modern basketball. The multiple change-of-direction (COD) dribbling and layup test captures three key dimensions: change-of-direction agility, non-steady-state ball-handling coordination, and resistance to interference, realistically simulating in-game demands such as fast-break transitions and driving to penetrate defenses. Compared to isolated laboratory metrics, this set of test tasks is deeply rooted in real basketball contexts. Their compound stress characteristics are more sensitive to training interventions and can be directly translated into potential improvements in on-court performance. Therefore, this testing system not only meets the standardized requirements of the national sports-specific entrance examination—ensuring that the research results are directly comparable with athlete grading systems and university admission criteria—but also, through the integration of multi-dimensional movement chains, achieves an ecological analysis of the structure of basketball-specific performance in basketball players. It thus provides an official, standardized reference system for evaluating training effectiveness.

##### 2.4.3.2 Vertical jump

Method: Maximum vertical jump reach with a running one- or two-foot takeoff. Height reached was recorded. Two attempts were given, with the best score used.

##### 2.4.3.3 Shooting

**Figure 1:**
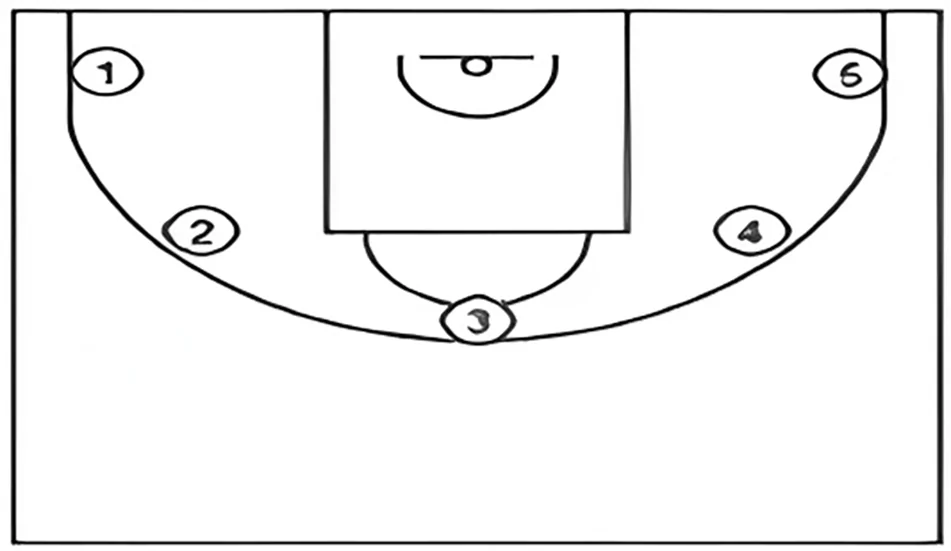
Court layout for the shooting test.

Method: As illustrated in Figure 1, five shooting spots were established outside an arc with a radius of 5.5 meters drawn from the projection point of the basket's center. These spots included the two 0° corners, the two 45° wings, and the top of the key (center of the arc). Four basketballs were placed at each spot, totaling 20 balls. Participants were required to begin shooting from either Spot 1 or Spot 5 and proceed to shoot all four balls at each consecutive spot, moving in either a clockwise or counterclockwise direction. For the fourth ball at each spot, participants had the option to shoot from behind the three-point line. The total test duration was one minute. All shots had to be taken from outside the arc; a made basket was considered invalid if either foot was on or over the line at the moment of release. Each participant performed two trials, with the best score (highest number of successful shots) recorded.

##### 2.4.3.4 Multiple change-of-direction dribbling and layup

**Figure 2:**
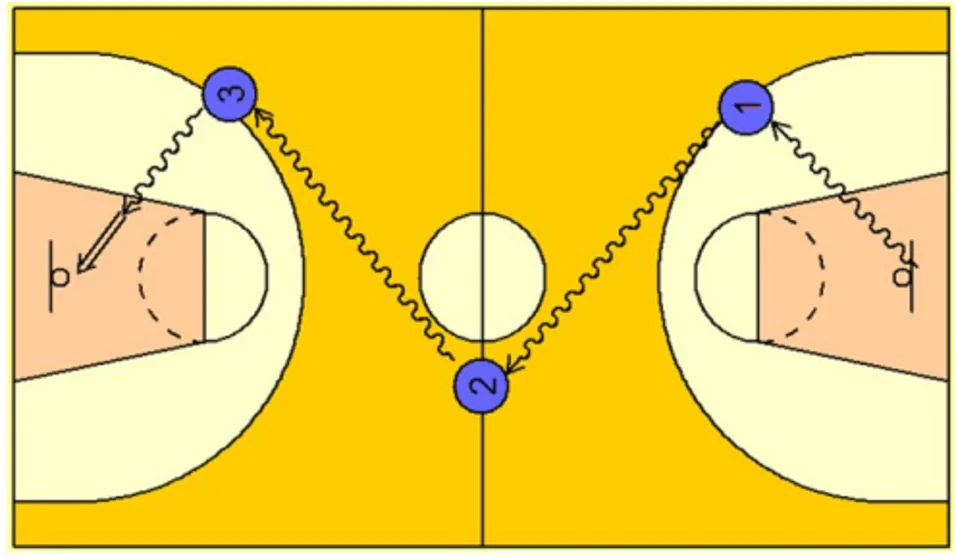
Schematic diagram of the multiple change-of-direction dribbling layup test.

Method: As shown in Figure 2, the athlete stands with the ball in the starting area outside the midpoint of the baseline. Timing begins when any part of the athlete's body breaks the vertical plane of the outer edge of the baseline. The athlete dribbles with the right hand to point ①, executes a right-hand behind-the-back dribble at ①, switches to the left hand and dribbles to point ②. At point ②, the athlete performs a left-hand spin dribble, switches to the right hand, and dribbles to point ③. At point ③, the athlete executes a right-hand between-the-legs dribble followed by a right-handed layup. After the ball goes through the basket, the athlete may use the left hand to dribble back to point ③. At point ③, a left-hand behind-the-back dribble is performed, followed by a switch to the right hand to dribble to point ②. At point ②, a right-hand spin dribble is performed, followed by a switch to the left hand to dribble to point ①. At point ①, a left-hand between-the-legs dribble is executed, followed by a left-handed layup. After this basket is made, the entire sequence (from the initial start to this point) is repeated once more. After the final layup is made, the athlete dribbles across the baseline. Timing stops when any part of the athlete's body breaks the vertical plane of the outer edge of the baseline. The total completion time is recorded. Each athlete performs two trials, and the best (fastest) time is used.

The markers ①, ②, and ③ on the basketball court are circles with a radius of 40 cm. The distance from the center of circles ① and ③ to the inner edge of the baseline is 6 meters, and the distance to the inner edge of the sideline is 2 meters. Marker ② is located on the midcourt line, with its center 2.8 meters from the center of the center circle. During the test, the athlete must have at least one foot on the line or inside the area of the relevant circle (①, ②, or ③) to perform the designated dribble move; otherwise, the attempt is considered invalid and no score is recorded. All layups must be made successfully. If a layup is missed and the athlete continues, the attempt is invalid and no score is recorded. Any violation (e.g., traveling, double dribble) during the dribbling sequence will result in a 1-second penalty added to the final time for each occurrence. The athlete must use the specified hand for each layup; using the wrong hand results in a 1-second penalty per occurrence. During between-the-legs dribbles, the ball must be moved from the front of the body from the inside to the outside to change hands, and both feet must remain on the ground. Failure to do so results in a 1-second penalty per occurrence.

FMS and McGill core stability tests in this study were administered by two assessors, each holding a master's degree or higher in Kinesiology (Human Movement Science). The two assessors were assigned different tests and performed the evaluations independently throughout the entire process, each scoring separately. All assessors completed systematic, specialized training on the FMS scoring criteria and the McGill test procedures, possessing solid theoretical knowledge and extensive practical experience in movement function assessment, thereby ensuring the professionalism of the evaluation. Prior to formal testing, the research team organized a unified scoring calibration session for all assessors to standardize the judgment criteria and minimize subjective bias. Given the inherent subjectivity of FMS scoring, the standardized training and scoring calibration in this study resulted in good inter-rater consistency, effectively controlling subjective assessment error. For the McGill core stability tests, a pilot study confirmed ideal test-retest reliability and inter-rater reliability, with standardized procedures and stable outcomes, meeting the requirements for core stability measurement in this study.

### 2.5 Statistical analysis

G*Power 3.1.9.7 was employed to estimate the sample size for this study. Data analysis was conducted using SPSS 26.0 and GraphPad Prism 9.0. First, descriptive statistics were calculated to determine the means and standard deviations of all variables and to describe their distribution characteristics, while also testing the normality and homogeneity of variance. Subsequently, Pearson product-moment correlation analysis was used to examine the linear relationships among core stability, FMS, and athletic performance indicators. The significance level was set at *P* < 0.05 and *P* < 0.01. Finally, stepwise multiple linear regression analysis was performed to evaluate the independent predictive effects of core stability and functional movement quality on different athletic performance measures.

## 3 Results and analysis

### 3.1 Descriptive analysis of participant characteristics

This study strictly adhered to the inclusion and exclusion criteria, recruiting a total of 33 qualified male university basketball players. The participants’ mean training duration was 5.18 ± 0.84 years, and their mean basketball experience was 6.70 ± 0.91 years, as shown in Table 2. To assess the representativeness of the sample, these training and experience indicators were compared with publicly available data for the population of Chinese national-level male university basketball players. An independent samples t-test revealed a statistically significant difference (t = 1.72, *P* = 0.003), as shown in Table 3, indicating that the training duration of the study sample falls within the typical range for the population of national-level male university basketball players.

To further verify the rationale for the adopted sample size, a *post-hoc* power analysis was conducted using G*Power 3.1.9.7 software, with “total athletic performance score” as the primary outcome measure. Based on a two-sided independent samples t-test, the effect size (Cohen's d) calculated from the actual data was 0.65, indicating a medium-to-large effect. The specific results are shown in Table 5. The results indicate that with a total sample size of *N* = 33, the statistical power (1-*β*) reached 0.89, exceeding the recommended threshold of 0.8. Additionally, the lower limit of the confidence interval for the effect size was above the small effect threshold. This suggests that the current sample size is sufficient to detect meaningful functional differences, confirming the sample size is reasonable.

**Table 5:** G*power analysis results.

Output indicator	Numerical value	Interpretation
Statistical Power (1-*β*)	0.89	Significantly higher than the recommended threshold of 0.8 in the field
Critical t-value	±2.042	Meets the critical value requirement for a two-tailed test with *α*=0.05
Confidence Interval for Effect Size	[0.21,1.08]	The lower limit still exceeds the threshold for a small effect size (0.2)
Total Sample Size Required	28 participants	The current sample of 33 participants meets the requirement

### 3.2 Correlation between athletic performance and FMS

As shown in the correlation analysis between performance measures and functional movements in Table 6, for the vertical jump test, the hurdle step, shoulder mobility, trunk stability push-up, and the total FMS score exhibited significant correlations with vertical jump performance. Among these, the hurdle step, trunk stability push-up, and total FMS score demonstrated highly significant positive correlations with vertical jump. For the shooting test, only rotary stability showed a significant negative correlation with shooting accuracy. For the multiple COD dribbling and layup test, the active straight leg raise displayed a highly significant negative correlation with performance, as detailed in Table 6.

**Table 6:** Correlations between performance measures and FMS components.

Projects	Vertical Jump		Shooting		Multiple COD dribbling and layup	
	R	P	R	P	R	P
Deep Squat	0.279	0.116	−0.151	0.403	0.151	0.402
Hurdle Step	0.472	0.006**	0.247	0.166	−0.210	0.240
Inline Lunge	0.156	0.385	−0.081	0.654	−0.317	0.072
Shoulder Mobility	0.359	0.040*	0.170	0.344	0.053	0.769
Active Straight Leg Raise	0.271	0.127	−0.040	0.827	−0.471	0.006**
Trunk Stability Push-up	0.452	0.008**	−0.003	0.989	0.101	0.578
Rotary Stability	0.083	0.647	−0.347	0.048*	0.056	0.755
Total Score	0.601	＜0.001**	−0.031	0.863	−0.164	0.363

*P < 0.05 indicates a significant correlation, **P < 0.01 indicates a highly significant correlation.

### 3.3 Relationship between athletic performance and core stability

As shown in the correlation analysis between performance measures and core stability tests in Table 7, for the vertical jump test, left trunk endurance, right trunk endurance, and back extensor endurance exhibited significant correlations with vertical jump performance. For the multiple COD dribbling and layup test, back extensor endurance showed a significant positive correlation with performance.

**Table 7:** Correlations between athletic performance and core stability.

Projects	Vertical Jump		Shooting		Multiple COD Dribbling and Layup	
	R	P	R	P	R	P
Left Trunk Endurance	−0.383	0.028*	−0.316	0.073	0.013	0.943
Right Trunk Endurance	0.346	0.048*	−0.104	0.564	0.342	0.051
Trunk Flexor Endurance	0.080	0.659	0.050	0.783	−0.069	0.701
Back Extensor Endurance	0.350	0.046*	−0.248	0.164	0.478	0.005**

*P < 0.05, **P < 0.01 indicate significant and highly significant correlations, respectively.

### 3.4 Relationship between core stability and FMS

As shown in Table 8, none of the core stability indicators demonstrated statistically significant correlations with any of the Functional Movement Screen (FMS) tests or the total FMS score (*P* > 0.05). Specifically, the *P*-values corresponding to the correlation coefficients between left trunk endurance, right trunk endurance, trunk flexor endurance, and trunk back extensor endurance and the FMS items—including the Deep Squat, Hurdle Step, Inline Lunge, Shoulder Mobility, Active Straight Leg Raise, Trunk Stability Push-up, Rotary Stability—as well as the total FMS score were all greater than 0.05. This indicates that no significant correlation exists between core stability and functional movement quality.

**Table 8:** Correlations between core stability and FMS.

Projects	Left Trunk Endurance		Right Trunk Endurance		Trunk Flexor Endurance		Back Extensor Endurance	
	R	P	R	P	R	P	R	P
Deep Squat	0.048	0.791	0.321	0.069	−0.050	0.783	0.339	0.054
Hurdle Step	−0.080	0.657	0.334	0.058	0.017	0.925	0.110	0.544
Inline Lunge	−0.046	0.798	0.107	0.555	−0.247	0.166	−0.138	0.444
Shoulder Mobility	−0.334	0.058	−0.020	0.914	−0.341	0.052	0.270	0.129
Active Straight Leg Raise	−0.091	0.614	−0.089	0.623	0.051	0.793	−0.272	0.126
Trunk Stability Push-up	−0.097	0.593	0.236	0.186	−0.059	0.745	0.178	0.321
Rotary Stability	−0.003	0.985	0.186	0.300	0.022	0.903	0.142	0.432
Total Score	−0.205	0.253	0.270	0.128	−0.120	0.505	0.196	0.274

*P < 0.05, **P < 0.01 indicate significant and highly significant correlations, respectively.

## 4 Discussion

This study investigated the correlations among core stability, functional movement, and athletic performance in male university basketball players. The results revealed specific and differentiated association patterns between different performance measures and the various FMS and core stability indicators.

### 4.1 Correlation between functional movement and athletic performance

The findings indicate that the Functional Movement Screen exhibits specific and differential associations with basketball performance measures. Overall, vertical jump performance, representing integrated explosive power, showed significant positive correlations with multiple FMS components and the total score. In contrast, the more complex technical skills of shooting and change-of-direction dribbling were associated with only isolated FMS items.

#### 4.1.1 Correlation between vertical jump and FMS

Vertical jump performance showed significant positive correlations with the hurdle step, shoulder mobility, trunk stability push-up, and the total FMS score. (1) Hurdle step. This test demands coordination and center-of-mass control during unilateral dynamic stability. This ability influences the efficiency of force and impulse transmission upward during the approach phase of a vertical jump. Individuals with superior Hurdle Step performance likely possess enhanced dynamic coordination and control of the lower limbs, which helps optimize take-off mechanics. This aligns with the established association between Hurdle Step performance in the FMS and vertical jumping ability (7). (2) Shoulder mobility. Adequate joint range of motion allows for optimal biomechanical positioning—specifically vertical alignment and maximal reach—during the airborne phase of the jump, contributing to a higher point of contact. (3) Trunk stability push-up. This test assesses core stability during an open-chain upper-body movement, which may relate to the core stability and scapulothoracic synergy required for shooting actions (15). In the vertical jump, the trunk acts as a pivotal hub for force transfer. Its rigid stability at the moment of take-off is crucial for the effective transmission of lower-body force to the upper body, directly influencing jump height. The significant positive correlation found in this study supports this viewpoint and is consistent with FMS theory, which emphasizes the supportive role of core stability and movement patterns for sport-specific abilities (16, 17). (4)Total FMS score. This composite score reflects the overall quality of whole-body functional movement, including flexibility, stability, symmetry, and movement pattern proficiency. The vertical jump is a kinetic chain action involving coordinated force production from the ankles, knees, hips, core, and shoulder, elbow, and wrist joints. Its performance is inherently constrained by the quality of the overall movement pattern. The strong positive correlation with the total FMS score confirms that the holistic optimization of fundamental functional movement capacity forms a potential foundation for improving vertical jump performance (16, 18).

#### 4.1.2 Correlation between shooting and FMS

Shooting is a highly structured, complex skill that relies heavily on precise neuromuscular coordination and proprioception, involving lower-body drive, core control, and fine motor execution of the upper limbs. In this study, shooting performance showed a significant negative correlation only with Rotary Stability, while no other FMS components or the total score demonstrated significant associations. This suggests that for highly practiced technical skills, a global assessment of basic functional movement patterns may not serve as a sensitive or effective predictor of performance.

Notably, the negative correlation between Rotary Stability and shooting may reveal specific movement preferences or potential compensatory strategies employed by basketball players during highly specialized shooting execution (19, 20). For instance, to enhance release stability and resistance to defensive pressure, elite athletes may strategically minimize or control compensatory trunk rotation during the shot, which conflicts with the multi-planar trunk coordination required in the FMS Rotary Stability test. This also explains the weak overall association between shooting and the total FMS score, indicating that screening tools like the FMS, which assess fundamental, multi-faceted movement patterns, have limitations in predicting highly technical and well-established sport-specific skill performance.

#### 4.1.3 Correlation between multiple COD dribbling and FMS

The active straight leg raise (ASLR) primarily assesses active hip flexor flexibility and pelvic-core dynamic control in open-chain movement. A lower score indicates greater limitation in hip flexion flexibility. In this study, performance in the multiple COD dribbling and layup test showed a significant negative correlation with the ASLR, while no other FMS components or the total score demonstrated significant correlations. This finding aligns with the conclusions of Lockie et al. (21), suggesting three possibilities: (1) The specific multiple COD dribbling and layup test designed for this study may place relatively low demands on hip flexibility; (2) The FMS scoring system may lack sufficient sensitivity in detecting optimal ranges of motion in high-level athletes; or (3) In the complex dynamic task assessed in this study, the influence of other factors far outweighs the limiting effect of single-joint mobility. Regardless of which possibility holds true, it indicates that the predictive variables for athletic performance in demanding basketball dynamic skills, such as the multiple COD dribbling and layup, are complex, and a single FMS test cannot effectively capture the core influencing factors of such movements.

In summary, the predictive value and correlational strength of the FMS exhibit pronounced specificity within basketball performance. It demonstrates relatively good predictive utility for whole-body, coordinative explosive tasks such as the vertical jump, whereas its association with fine motor skills like shooting is weak, and more complex dynamic skills appear sensitive only to isolated mobility metrics. This indicates that the evaluative utility of the FMS in the basketball-specific context and its application for training guidance require careful analysis of both the direction and strength of its relationship with specific performance indicators.

### 4.2 Correlation between core stability and performance

Core stability, assessed via the McGill endurance battery, also showed complex and specific associations with basketball performance. Core endurance measures were more noticeably correlated with VJ and multiple COD dribbling but showed no significant links to shooting performance.

#### 4.2.1 Correlation between vertical jump and core endurance

VJ correlated significantly with left lateral trunk endurance, right lateral trunk endurance, and back extensor endurance. (1) Integration Mechanism:Core muscles act as a central hub for force transfer during jumping, providing a rigid base for the hip-spine complex to optimize the efficiency of lower limb force transmission upward (5) Back extensor endurance likely enhances the ability to maintain slight trunk extension during takeoff, creating a stable channel for force transfer, thereby supporting higher jump performance, consistent with the view that core endurance contributes positively to vertical jumping (22, 23) (1, 20), (2) Left-right asymmetry in endurance performance: Notably, core endurance exhibited opposing effects on the left and right sides, showing a positive correlation on the right side and a negative correlation on the left side. This phenomenon likely highlights the prevalent movement asymmetries and neuromuscular adaptation patterns inherent in basketball. The sport involves a high volume of actions oriented toward the basket, emphasizes right-limb dominance in force production, and requires frequent directional changes in the sagittal plane. These demands may lead to differences between the left and right core muscles in terms of strength requirements, activation patterns, and fatigue levels (9). Asymmetry in lower limb endurance performance is associated with risk factors for lower extremity overuse injuries, and its association pattern with athletic performance warrants further investigation. The results of this study suggest that a generalized discussion of “bilateral core endurance” in relation to athletic performance is insufficient, and attention should be paid to the specific association characteristics of endurance performance in different directions and at different sites. (3) Selective influence: Trunk flexor endurance showed no significant correlation with vertical jump height in this study, indicating that this directional endurance does not exhibit a clear covariation trend with vertical jumping performance, which primarily involves sagittal-plane force transfer.

#### 4.2.2 Correlation between shooting and core endurance

This study found no significant association between shooting performance and core stability, which aligns with possible differences in perspective from certain interventional studies (22). This discrepancy may arise from the fact that shooting is a highly technical and neuro-driven action, whereas the form of core endurance testing employed may not align with the physiological demands of the core functions required for shooting skills. The aggregate measures in this study may have conflated different core functional dimensions, such as strength, coordination, or neural control (8, 23).

No significant statistical association was found between shooting performance indicators and any of the core stability measures, which may be related to the complexity of the shooting action and a mismatch between the tests and actual demands. Firstly, shooting is a highly technical and neurally refined skill whose performance is primarily governed by multiple factors such as upper limb joint coordination, fine motor control of the wrist and fingers, proprioceptive stability, hand-eye coordination, and movement proficiency. In this action, the core muscles predominantly serve a foundational support role, with their primary task being to provide transient, short-term stabilization during the brief release phase and to facilitate appropriate tension transfer. Secondly, the McGill core endurance tests employed in this study were designed to assess muscular fatigue resistance during sustained isometric postures, which differs from the core functional demands of shooting that require “transient stability+static support+coordinated transfer.” Core endurance is only one dimension of core stability, while the core function relied upon in shooting may lean more toward isometric strength control in specific static postures, or its influence may have been “diluted” or “overshadowed” by the dominant factor of upper-limb precision. This contrasts with conclusions from other studies suggesting that core strengthening can improve shooting ability (22) and that core stability training can enhance shooting accuracy (8, 23). Such discrepancies may stem from differences in measurement objectives, training interventions, and sample characteristics across studies. The associations between endurance performance of different core muscle groups and basketball-specific performance exhibit clear performance-specific and muscle group-specific characteristics. Vertical jump performance was significantly correlated with left-side, right-side, and back extensor endurance, with different correlation patterns, suggesting specific covariation trends between left-right asymmetry in endurance levels, back extensor endurance characteristics, and vertical jump performance.

High-intensity dynamic composite skills like change-of-direction dribbling strongly depend on the fatigue resistance of the back extensors, which serve as the “foundation” for maintaining core rigidity and ensuring efficient energy transfer during high-speed movements. Conversely, precision-oriented technical performances such as shooting appear “decoupled” from core endurance measures, reflecting a unique bias in core functional demands imposed by technical complexity. The quantitative analysis of these association patterns guides coaches in identifying and specifically strengthening the particular core endurance dimensions that exert the greatest leverage on different performance outcomes, thereby providing critical direction for the precise training of basketball athletes.

#### 4.2.3 Correlation between multiple COD dribbling and core endurance

Multiple COD dribbling performance showed a positive correlation with back extensor endurance and a trend with right-side endurance. The strong positive correlation with back extensor endurance is the most specific and robust finding regarding core endurance, aligning with research showing core stability training improves agility (6). It strongly indicates that the fatigue resistance of the back extensors is a key physiological foundation for efficient, rapid directional changes. During multiple COD and layups, athletes must rapidly adjust their center of mass against external perturbations. Strong back extensor endurance helps maintain trunk stability under high-speed, off-balance conditions, preventing energy loss or movement breakdown due to core “collapse,” thereby ensuring effective execution of lower-limb agility and upper-limb finishing skills (9, 24). This also highlights a potential limitation of the FMS, which assesses “instantaneous” stability and may not sensitively reflect the core's (especially the back's) ability to maintain function under sustained load, a common demand in basketball.

### 4.3 Correlation between functional movement and core stability

The study found no significant statistical correlations between core stability (as characterized by McGill endurance tests) and functional movement ability (as characterized by FMS) in this cohort of university basketball players. This result challenges some theoretical expectations of integration and has important implications for theory and training assessment.

First, Table 2 clearly shows that all core endurance measures were uncorrelated with FMS components and the total score. This finding is not isolated, as previous studies have also reported a lack of systematic association between core endurance tests and FMS scores contrasting with conclusions suggesting a close link between core stability and functional movement patterns in basketball (25, 26).

Secondly, the fundamental differences in core concepts and measurement dimensions are the root cause of this result, as the two assessments target distinct dimensions of physical function and employ markedly different measurement paradigms. Specifically, the McGill core stability tests focus on the fatigue resistance of specific core muscle groups during sustained isometric postures. The results reflect the physiological exhaustion threshold of localized muscles under constant load, essentially using duration as a bio-measure of a muscle's capacity for sustained work. In contrast, the FMS is based on the kinetic chain concept, evaluating the quality of fundamental movement patterns during specific, low-repetition, non-externally loaded test actions. Its core focus lies in the symmetry, coordination, flexibility, and movement stability control strategies of the body. The scores reflect the overall neuromuscular efficiency in coordination or stability demonstrated during these specifically designed movement screens. Therefore, each assessment essentially targets deep structural indicators within its respective domain, and within the framework of this study, they demonstrate statistical independence.

In summary, the findings revealed in this study provide substantial support for coaches and researchers when designing performance enhancement plans for basketball athletes. Firstly, FMS scores should not be simplistically used as an inverse indicator of core stability, nor should endurance test durations alone be used to infer issues with dynamic movement control. Secondly, the mutual independence between functional movement and core stability necessitates distinct training emphases: neuromuscular integration drills emphasizing synergy and precise control should be prioritized for optimizing functional movement, while addressing localized weaknesses in core muscular endurance requires targeted protocols focusing on sustained static-dynamic capacity. This does not imply that the two systems are physiologically unrelated at a macro level. Rather, it underscores the necessity of acknowledging their dichotomous underlying dimensions and employing differentiated training methodologies, not that they lack structural synergy within the human torso's overall stabilization strategy. Within this assessment framework, the highly specialized functional evaluations exhibit a high degree of exclusivity, thus complementarity should be regarded as a foundational principle in combined assessment procedures.

In conclusion, core stability and functional movement quality, as measured in this context, represent independent distributional dimensions. This study supports the concurrent application of the FMS to screen for movement compensation and the McGill battery to measure core endurance indicators in university basketball athletes, with separate, targeted training prescriptions developed accordingly. Furthermore, this indicates that future scientific basketball training must carefully delineate the two training aspects of “functional movement” and “core stability,” while simultaneously developing domain-specific training progression systems, monitoring paradigms, and standards for integrated application.

## 5 Conclusion and practical applications

Through quantitative analysis, this study explored, for the first time in a population of male university basketball players, the association characteristics of functional movement quality and core stability as two independent capacity dimensions. The results showed that these two dimensions exhibited differentiated association patterns with different basketball-specific performance measures. The conclusions are as follows:

(1) The predictive value of functional movement quality for athletic performance exhibits significant sport-specificity.

The total Functional Movement Screen score, along with indicators such as the Hurdle Step and Trunk Stability Push-up, can effectively predict vertical jump performance, corroborating the supportive role of whole-body coordination and fundamental movement pattern optimization for vertical explosive power. However, its predictive value for performance in complex technical actions, such as shooting accuracy and change-of-direction dribbling efficiency, is limited. This suggests that the FMS has limitations in sensitivity when evaluating basketball-specific skills. In training, it should be positioned as a preliminary tool for screening fundamental compensations and optimizing movement patterns, rather than as a direct predictor of basketball-specific performance.

(2) This study found that core stability was strongly correlated with dynamic change-of-direction performance, and the association pattern exhibited muscle group specificity and left-right asymmetry.

Trunk back extensor endurance is a strong indicator for predicting performance in the multiple COD dribbling and layup test, confirming the core role of fatigue-resistant back extensors in maintaining trunk rigidity during high-speed directional changes and ensuring efficient force transfer. Core endurance demonstrates a left-right asymmetric effect, suggesting that neuromuscular adaptations resulting from basketball activities may influence force transfer efficiency, a key consideration for both performance enhancement and injury prevention. Notably, no significant correlations were found between any dimensions of core stability and shooting performance, indicating that static endurance tests may not adequately capture the core functional characteristics required for finely coordinated technical actions like shooting.

In the practical application of sports training and injury prevention, training pathways should be designed separately according to the biomechanical demands of target actions: optimizing functional movement patterns versus enhancing specific core muscle group fatigue resistance. This approach avoids non-specific, generalized “core training,” thereby maximizing training efficiency and minimizing potential risks. For explosive actions like the vertical jump, priority should be given to using the FMS to assess and correct symmetry and coordination deficits in fundamental movement patterns (e.g., Deep Squat, Hurdle Step, Trunk Stability Push-up). This optimizes neuromuscular control and the efficiency of the force transfer chain, laying the foundation for explosive performance. For change-of-direction actions involving dynamic opposition and high impact, the training focus should be on strengthening the fatigue resistance of the trunk extensor muscles and specifically correcting left-right imbalances in core endurance. This ensures sufficient trunk rigidity and stability during high-speed, high-intensity movements, effectively reducing injury risk.

## 6 Limitations and research prospects

The main limitations of this study should be considered when interpreting the results and applying them in practice. First, the relatively small sample size and cross-sectional observational design limit the ability to infer causality and preclude in-depth subgroup analyses, affecting statistical power and the generalizability of the conclusions. Second, the conclusions of this study are only applicable to male college basketball players aged 18–25 years, as the study was limited to participants from a single university and only included male collegiate basketball players, lacking representation from adolescents, professional athletes, female athletes, and other sports. This restricts the generalizability of the findings to broader competitive levels, genders, or sports populations. Finally, the measurement tools have functional limitations. As an assessment of fundamental movement patterns, the FMS does not fully integrate the complexity of basketball-specific techniques, leading to insufficient sensitivity for core skills like shooting and high-intensity directional changes. The core stability tests focused solely on the single dimension of muscular endurance, failing to encompass crucial functional demands of actual competition, such as dynamic core stability, eccentric control, and rotation.

Future research should aim to expand the sample size and employ prospective cohort or randomized controlled designs. Broadening the population coverage would enhance representativeness. Furthermore, developing functional assessment tools that integrate the technical characteristics of basketball, incorporating core stability indicators such as dynamic anti-rotation and rapid response, is necessary. Longitudinal intervention studies should be conducted to empirically test the efficacy of “dissociated” training programs in enhancing performance and reducing injury risk.

## Data Availability

The original contributions presented in the study are included in the article/Supplementary Material, further inquiries can be directed to the corresponding author.

## References

[1] Li HY. Core strength training influences basketball Players’ body. Rev Bras Med Esporte. (2022) 28(6):654–7. 10.1590/1517-8692202228062022_0031

[2] Lu Y Li W Li J. Influence of functional training on special technical movement ability and athletic performance of male basketball players. Rev Psicol Deporte. (2023) 32(4):94–108.

[3] Luo S Soh KG Soh KL Sun H Nasiruddin NJM Du C. Effect of core training on skill performance among athletes: a systematic review. Front Physiol. (2022) 13:915259. 10.3389/fphys.2022.915259 35755428 PMC9227831

[4] Cook G Burton L Hoogenboom B. Pre-participation screening: the use of fundamental movements as an assessment of function - part 2. N Am J Sports Phys Ther: NAJSPT. (2006) 1(3):132–9. , PMCID: PMC2953359 21522225 PMC2953359

[5] Xing QJ Shen YY Cao R Zong SX Zhao SX Shen YF. Functional movement screen dataset collected with two azure kinect depth sensors. Sci Data. (2022) 9(1):104. 10.1038/s41597-022-01188-7 35338164 PMC8956653

[6] Li Y. Strengthening the abdominal core on balance in basketball players. Revista Brasileira de Medicina do Esporte. (2023) 29:e2022–0598. 10.1590/1517-8692202329012022_0598

[7] Gonzalo-Skok O Serna J Rhea MR Marín PJ. Relationships between functional movement tests and performance tests in young elite male basketball players. Int J Sports Phys Ther. (2015) 10(5):628–38. PMID: 26491613, PMCID: PMC4595916 26491613 PMC4595916

[8] Silfies SP Ebaugh D Pontillo M Butowicz CM. Critical review of the impact of core stability on upper extremity athletic injury and performance. Braz J Phys Ther. (2015) 19(5):360–8. 10.1590/bjpt-rbf.2014.0108 26537806 PMC4647147

[9] Wilkerson GB Giles JL Seibel DK. Prediction of core and lower extremity strains and sprains in collegiate football players: a preliminary study. J Athl Train. (2012) 47(3):264–72. 10.4085/1062-6050-47.3.17 22892407 PMC3392156

[10] Garbenytė-Apolinskienė T Šiupšinskas L Salatkaitė S Gudas R Radvila R. The effect of integrated training program on functional movements patterns, dynamic stability, biomechanics, and muscle strength of lower limbs in elite young basketball players. Sport Sci Health. (2017) 14(2):245–50. 10.1007/s11332-017-0409-y

[11] Lee S Kim H Kim J. The functional movement screen total score and physical performance in elite male collegiate soccer players. J Exerc Rehabil. (2019) 15(5):657–62. 10.12965/jer.1938422.211 31723553 PMC6834696

[12] Harshbarger ND Anderson BE Lam KC. Is there a relationship between the functional movement screen, star excursion balance test, and balance error scoring system? Clin J Sport Med. (2018) 28(4):389–94. 10.1097/JSM.0000000000000465 28742602

[13] Fitton Davies K Sacko RS Lyons MA Duncan MJ. Association between functional movement screen scores and athletic performance in adolescents: a systematic review. Sports (Basel). (2022) 10(3):28. 10.3390/sports10030028 35324637 PMC8954950

[14] O’Brien W Khodaverdi Z Bolger L Tarantino G Philpott C Neville RD. The assessment of functional movement in children and adolescents: a systematic review and meta-analysis. Sports Med. (2022) 52(1):37–53. 10.1007/s40279-021-01529-3 34524655 PMC8761122

[15] Zemková E Zapletalová L. The role of neuromuscular control of postural and core stability in functional movement and athlete performance. Front Physiol. (2022) 13:796097. 10.3389/fphys.2022.796097 35283763 PMC8909639

[16] Cook G Burton L Hoogenboom B. Pre-participation screening: the use of fundamental movements as an assessment of function - part 1. N Am J Sports Phys Ther: NAJSPT. (2006) 1(2):62–72. PMID: 21522216, PMCID: PMC2953313 21522216 PMC2953313

[17] Zhang M Miao X Rupčić T Sansone P Vencúrik T Li F. Determining the relationship between physical capacities, metabolic capacities, and dynamic three-point shooting accuracy in professional female basketball players. Appl Sci. (2023) 13:8624. 10.3390/app13158624

[18] Canlı U Koçak ÇV. The relationship of shooting skill with functional movement performance and attention level of basketball players. J Educ Train Stud. (2019) 6(12a). 10.11114/jets.v6i12a.3926

[19] A literature review of the functional movement screen as a predictor of injury in the sport of basketball.

[20] Sannicandro I Cofano G. Core stability training and jump performance in young basketball players. Int J Sci Res (IJSR). (2017) 6(5):479–82. 10.21275/ART20173282

[21] Lockie R Schultz A Callaghan S Corrin C Luczo T Jeffriess M. A preliminary investigation into the relationship between functional movement screen scores and athletic physical performance in female team sport athletes. Biol Sport. (2015) 32(1):41–51. 10.5604/20831862.1127281 25729149 PMC4314603

[22] Li X Liu H Lin KY Miao P Zhang BF Lu SW. Effects of different sling settings on electromyographic activities of selected trunk muscles: a preliminary research. Biomed Res Int. (2020) 2020:2945952. 10.1155/2020/2945952 31998786 PMC6970500

[23] Warren M Lininger M Chimera N Smith C. Utility of FMS to understand injury incidence in sports: current perspectives. Open Access J Sports Med. (2018) 9:171–82. 10.2147/OAJSM.S149139 30233259 PMC6135213

[24] Sannicandro I Cofano G Piccinno A. Can the core stability training influences sprint and jump performances in young basketball players? Adv Phys Educ. (2020) 10(03):196–206. 10.4236/ape.2020.103017

[25] Leetun DT Ireland ML Willson JD Ballantyne BT Davis IM. Core stability measures as risk factors for lower extremity injury in athletes. Med Sci Sports Exerc. (2004) 36(6):926–34. 10.1249/01.MSS.0000128145.75199.C3 15179160

[26] Okada T Huxel KC Nesser TW. Relationship between core stability, functional movement, and performance. J Strength Cond Res. (2011) 25(1):252–61. 10.1519/JSC.0b013e3181b22b3e 20179652

